# Presence of antigen-specific somatic allelic mutations and splice variants do not predict for immunological response to genetic vaccination

**DOI:** 10.1186/2051-1426-1-2

**Published:** 2013-05-29

**Authors:** Jordan T Becker, Douglas G McNeel

**Affiliations:** 1 Department of Medicine, University of Wisconsin Carbone Cancer Center, 1111 Highland Avenue, Madison, WI 53705, USA

**Keywords:** PAP, DNA vaccines, Alternative splice variants, Allelic variants

## Abstract

**Background:**

Antigen-specific anti-tumor vaccines have demonstrated clinical efficacy, but immunological and clinical responses appear to be patient-dependent. We hypothesized that naturally-occurring differences in amino acid sequence of a host’s target antigen might predict for immunological outcome from genetic vaccination by presentation of epitopes different from the vaccine.

**Methods:**

Using peripheral blood cells from 33 patients who had been treated with a DNA vaccine encoding prostatic acid phosphatase (PAP), we sequenced the exons encoding PAP and PSA genes from somatic DNA to identify single nucleotide polymorphisms. In addition, mRNA was collected to detect alternative splice variants of PAP.

**Results:**

We detected four synonymous coding mutations of PAP among 33 patients; non-synonymous coding mutations were not identified. Alternative splice variants of PAP were detected in 22/27 patients tested. The presence of detectable splice variants was not predictive of immunological outcome from vaccination. Immune responses to peptides encoded by these splice variants were common (16/27) prior to immunization, but not associated with immune responses elicited with vaccination.

**Conclusions:**

These results suggest that antigen-specific immune responses detectable after treatment with this genetic vaccine are specific for the host-encoded antigen and not due to epitope differences between the vaccine and a particular individual’s somatic coding sequence.

## Background

Immunotherapies, and antigen-specific vaccines in particular, are of great interest as a means of providing a tumor-specific therapy with ideally minimal toxicity. The recent successes with the 2010 FDA approval of Provenge™ and ipilimumab as treatments for metastatic prostate cancer and melanoma to prolong survival underscore the potential of these types of novel therapies [1,2]. In addition, in 2010 the USDA approved the canine melanoma vaccine, Oncept™, a DNA vaccine encoding human tyrosinase, on the basis of clinical trials demonstrating improved overall survival of dogs with melanoma treated with this vaccine [3]. A number of cancer immunotherapeutic vaccines for other malignancies have progressed to Phase II and Phase III clinical trials on the basis of demonstrable clinical benefit [4]. For example, data from a large Phase II trial of a viral vaccine encoding prostate-specific antigen, PROSTVAC™, suggested an increase in overall survival of patients with metastatic prostate cancer [5]. A similar genetic vaccine targeting MUC1 (TG4010) has been shown to enhance the effects of chemotherapy in a Phase IIb clinical trial of patients with advanced non-small cell lung carcinoma (NSCLC) and this vaccine is now in a Phase IIb/III clinical trial evaluating first-line safety and clinical efficacy in prolonging survival [6]. In trials using these antigen-specific vaccines investigators have sought to identify antigen-specific immune responses as possible biomarkers for those individuals likely to benefit clinically from vaccination. However, it has been observed that immunological responses are present in some patients and not in others, and that immunological response is not always associated with clinical response. Accordingly, some investigators examining cancer immunotherapies in preclinical and early Phase I and II clinical trials have chosen to utilize strategies meant to boost the frequency of immunological responders and/or broaden the durability and quality of the immune responses elicited. Current strategies under investigation include alternative means of vaccine delivery, use of adjuvants and/or combination therapies, use of multiple antigens and/or multiple vaccine vectors, and taking advantage of xenoantigen cross-reactivity [7-12].

Studies from our group and others highlight that DNA vaccines offer an off-the-shelf, inexpensive, and mutable therapeutic option with very minimal toxicity and broadly augmentable clinical applications as far as schedule and dosing are concerned [7]. Our work with a DNA vaccine targeting prostatic acid phosphatase (PAP) has shown clinical safety and immunological efficacy, eliciting durable PAP-specific T-cell responses capable of amplification by subsequent booster immunizations [13,14]. Other antigens currently being targeted by genetic vaccines in human clinical trials include: carcinoma-embryonic antigen (CEA), gp100, MAGE-A3, MUC1, NY-ESO-1, prostate-specific antigen (PSA), and prostate-specific membrane antigen (PSMA) [6,11,15-19].

Antigens encoded by genetic vaccines generally consist of a native DNA sequence based on readily available data from genomic and protein databases. Accordingly, xenogeneic vaccination utilizes highly homologous antigens specific to other species to hopefully elicit immune responses that cross-react with areas of similar or conserved epitopes. Xenogeneic cross-reactivity has specifically been researched and cited as an effective means of overcoming self-tolerance to tumor-associated antigens (TAAs) [12]. Indeed, the USDA approval of Oncept™ (Merial Ltd, Duluth, GA, USA) helped to highlight the immunological and clinical success of xenogeneic vaccination, specifically the treatment of canine melanoma with a DNA vaccine encoding human tyrosinase [3]. Xenogeneic vaccination, specifically DNA vaccines using prime-boost strategies targeting mouse and human PSMA, have been under investigation for prostate cancer [12]. Recent work from our laboratory has indicated that Lewis rats immunized with DNA vaccine encoding human PAP generate responses to the highly homologous human antigen; in fact, these responses recognize a single epitope that differs from the rat PAP sequence by only two amino acids. However, in this particular system there is no evidence of cross-reactivity to the corresponding rat epitope [20]. Conversely, Copenhagen rats immunized with vaccinia vector encoding human PAP generate cytotoxic T lymphocyte (CTL) responses and subsequently experience prostatitis, indicative of an immune response against rat PAP [21]. These results suggest that xenoantigens may or may not elicit productive anti-tumor immune responses, and the effects might be dependent upon the MHC type of the host. Analogously, we speculated that if a DNA vaccine encoding a standard antigen sequence was administered to patients who actually expressed a variation of that antigen, the vaccine-encoded-antigen (or some epitopes encoded) might be recognized as “foreign” in those patients. Thus, we reasoned that single nucleotide polymorphisms (SNPs) in the somatic PAP genes of patients might represent very highly homologous antigens and might be an avenue for enhanced immune response following antigen-specific vaccination, or alternatively might explain why immune responses occur, detectable to the homologous-but-foreign antigen, that are not associated with clinical response.

Using available online database resources we observed that others have previously detected SNPs among individuals’ genes encoding PAP (Ensembl Online Database). One previously detected SNP found among these data occurs in p299-307, a previously defined HLA-A2-restricted epitope of human PAP protein [22,23]. Additionally, a number of alternative splice variant messenger RNA transcripts exist for PAP, notably three protein-coding alternative splice variant transcripts. Allelic variants in the somatic gene encoding PAP, as well as preferentially-expressed alternative splice variants of PAP, may represent very highly homologous antigens distinct from the vaccine-encoded antigen. In the current report we sought to determine the frequency of PAP target gene SNPs and the frequency of alternative splice variants of PAP in our patient population, as well as their associations with immune response following vaccination.

## Results

### Amino acid coding mutations have been previously detected among common anti-tumor genetic vaccine antigens

Our laboratory and others have investigated anti-tumor genetic vaccines targeting a variety of protein antigens in clinical trials of breast cancer, gastrointestinal malignancy, melanoma, non-small cell lung cancer, ovarian cancer, and prostate cancer. Allelic variants have been detected for many of the common anti-tumor genetic vaccine antigens. Prepared using online resources, Table 1 summarizes the size and chromosomal location of genes encoding several common target antigens and highlights the frequency of previously identified SNPs in these genes. Notably, SNPs have been previously detected in the gene encoding PAP, the target of FDA-approved sipuleucel-T (Provenge™, Dendreon Corp., Seattle, WA, USA) as well as the target of a DNA vaccine currently being investigated in a Phase II clinical trial [1]. We included a “mutation quotient” in Table 1, defined as the sum of all exonic non-synonymous, stop loss/gain, and frameshift coding mutations, mutations that could specifically affect MHC-presented epitopes, for each gene divided by the amino acid (aa) length of the protein product. We observed that while some common vaccine target antigens are highly conserved (e.g. NY-ESO-1), others are not. For instance, 41 amino acid coding mutations that could affect presented epitopes have been detected in the gene encoding PSA (261 aa protein), whereas in the gene encoding PAP (386 aa) only 25 amino acid coding mutations have been detected. We focused these experiments on PAP as it is the target antigen of the FDA-approved sipuleucel-T and given that we have previously detected PAP-specific immune responses in a subset of patients receiving a genetic vaccine, pTVG-HP, encoding this antigen.

**Table 1:** Human genes encoding common target antigens of genetic vaccines show previously detected allelic variations

**Target antigen**	**AR**	**CEA**	**gp100**	**MAGE-A3**	**NY-ESO-1**	**PAP**	**PSA**	**PSMA**	**TYR**
Gene ID	AR-001	CEACAM5-001	PMEL-001	MAGEA3-001	CTAG1B-001	ACPP-001	KLK3-201	FOLH1-001	TYR-001
Location	Xq12	19q13.1-13.2	12q13-q14	Xq28	Xq28	3q21-q23	19q13.41	11p11.2	11q14-q21
Synonymous Coding Mutations	26	28	22	16	0	13	20	19	24
Non-Synonymous Coding Mutations	71	61	46	43	1	22	39	38	95
Stop Gain/Loss	6	1	2	0	0	3	0	0	9
Frameshift	1	1	0	0	0	0	2	2	13
Coding Unknown	337	0	0	0	0	0	0	1	207
Total	441	91	70	59	1	38	61	60	348
cDNA Length (bp)	10065	2907	2757	1724	998	3125	1464	2635	2466
Protein Length (aa)	920	702	661	314	168	386	261	750	529
**Mutation Quotient**	**0.085**	**0.090**	**0.073**	**0.137**	**0.006**	**0.065**	**0.157**	**0.053**	**0.221**

Eight genes encoding genetic vaccine target antigens under investigation in human clinical trials, and one gene encoding a prostate cancer target antigen of interest (AR), are listed in this table by antigen name as well as Gene ID taken from the USEast Ensemble online database. The NCBI-Gene and USEast Ensemble online databases provided information regarding chromosomal location of antigen gene, cDNA length (bp), and protein length (aa) as well as total number of allelic variations previously detected by others. Allelic variations are categorized into synonymous, non-synonymous, stop gain/loss, frameshift, or unknown coding mutations accordingly. Mutation quotient is defined as the sum of non-synonymous coding mutations, stop gain/loss, and frameshift mutations (mutations that could affect immunologically presented epitopes) divided by protein length (aa). Coding mutation online queries were performed April 16, 2012.

### Allelic variations in patient PAP gene are detectable but non-synonymous coding mutations are not associated with differences in PAP-specific immune responses

Our previously published reports indicate that multiple patients receiving anti-tumor vaccination against the self-antigen PAP showed evidence of antigen-specific immune responses detectable either post-vaccination or developing up to one year following immunization [13,14]. However, we failed to detect PAP-specific immune responses in some patients and additionally, in an on-going Phase Ib/II clinical trial using the same vaccine, we have observed similar differences in immune response development (unpublished data). We hypothesized that non-synonymous allelic variations in patient PAP gene could relegate vaccine-encoded PAP protein as essentially foreign, thereby increasing the immunogenicity of the DNA vaccine. Consequently, we sought to examine whether SNPs of the gene encoding PAP could be detected in the somatic DNA of patients receiving antigen-specific genetic vaccination targeting PAP. We then wished to determine whether patient immune response following antigen-specific vaccination was associated with detectable allelic variations in the target antigen, PAP.

**Figure 1:**
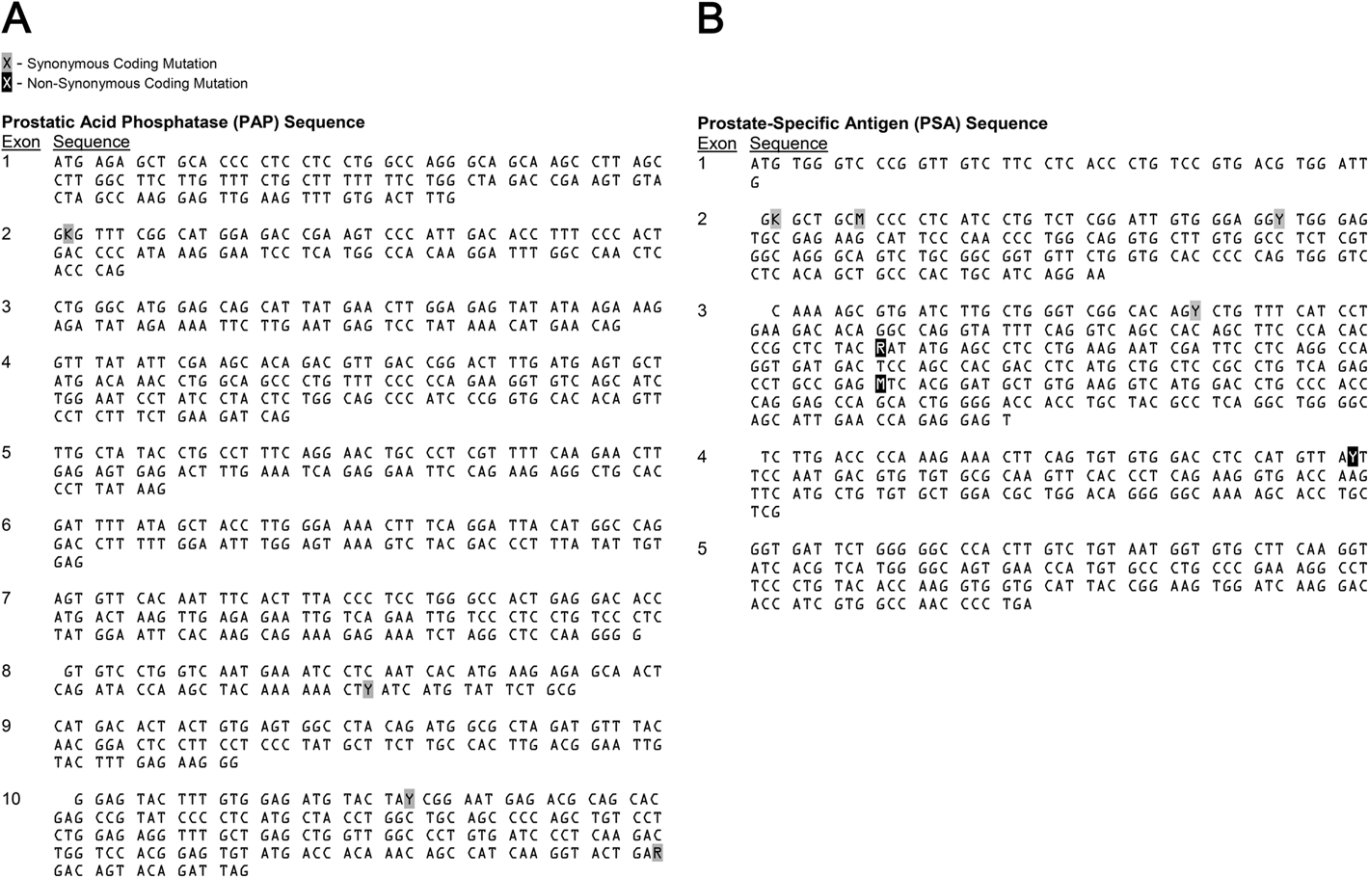
**Allelic variants in patient PAP and PSA genes are detectable.** Sanger sequencing was conducted to identify allelic variants in patients’ PAP gene (**A**) and PSA gene (**B**). Allelic variants are highlighted to indicate either synonymous (gray) or non-synonymous (black) coding mutations. Coding mutations are designated with transversions K (keto) and M (amino), as well as transitions R (purine), and Y (pyrimidine) IUPAC nucleotide ambiguity codes.

Using primers indicated in Additional file 1: Table S1, we sequenced genes encoding PAP from genomic DNA extracted from PBMC from a total of 33 patients. Among the ten exons of PAP, we detected four SNPs or allelic variants (Figure 1A). Six patients expressed a T>G transversion in the first codon of PAP exon 2 (PAP-c.122T>G, 6/33, 18.18%). Eleven patients expressed a C→T transition in the 23rd codon of PAP exon 8 (PAP-c.849C>T, 11/33, 33.33%). Fifteen patients expressed a T→C transition in the ninth codon of PAP exon 10 (PAP-c.993T>C, 15/33, 45.45%). Seventeen patients expressed an A→G transition in the 60th codon of PAP exon 10 (PAP-1146A>G, 17/33, 52.52%). As demonstrated in Table 2, we observed that the expression of PAP-c.122T>G differed significantly between immune responders and non-responders (*p=0.0015*, Fisher’s exact test). However, all of the PAP SNPs detected in our patients were synonymous coding mutations, producing no alterations in the amino acid sequence of the PAP protein. Thus, contrary to our hypothesis, patients with evidence of PAP-specific immune responses did not encode PAP protein different in amino acid sequence from the immunization vector-encoded PAP and it was therefore unlikely that immune responses detected following vaccination were due to responses to epitopes differing between the host and immunization vector.

**Table 2:** Patients express multiple allelic variations of PAP and PSA genes

		**PAP allelic variants**				**PSA allelic variants**						
	**Patients**	**122T>G**	**849C>T**	**993T>C**	**1146A>G**	**48T>G**	**54A>C**	**84C>T**	**237C>T**	**304G>A**	**394C>A**	**536T>C**
	1	**X**	**X**	**X**	**X**							***X***
	2	**X**	**X**	**X**	**X**							
	3	**X**								***X***		
	4	**X**										
	5	**X**										
	6	**X**										
Non-Responders	7		**X**	**X**	**X**							
	8		**X**	**X**	**X**							
	9									***X***		
	10											
	11											
	12											
	13		**X**	**X**	**X**							
	14											
	15											
	16			**X**	**X**							
	17		**X**	**X**	**X**			**X**				
	18			**X**	**X**							
	19		**X**	**X**	**X**				**X**		***X***	
	20			**X**	**X**							
	21			**X**	**X**							***X***
	22											
	23		**X**		**X**				**X**		***X***	
Immune Responders	24											
	25								**X**			
	26			**X**	**X**				**X**			
	27		**X**	**X**	**X**				**X**			
	28				**X**							
	29					**X**	**X**				***X***	
	30					**X**			**X**		***X***	
	31		**X**	**X**	**X**				**X**			
	32											
	33		**X**	**X**	**X**							

Shown are individual SNPs detected among PAP-specific immune non-responding patients (top, 1–13) and PAP-specific immune responding patients (bottom, 14–33). Detected allelic variants are named for their relative position within each gene such that PAP-c.122T>G is the T→G transversion in the 122 nucleotide from the cDNA start site. Detection of an allelic variant is indicated by an X. Bold face indicates synonymous coding mutations and bold face italic indicates non-synonymous coding mutations.

### Allelic variations in patient PSA gene are detectable and encode changes in amino acid sequence

Among these same 33 individual patient samples, we also sequenced patients’ genes encoding PSA as it is not only the target antigen of other genetic vaccines, PROSTVAC and a plasmid DNA vaccine, but is also the primary serum marker protein used in the diagnosis of prostate cancer and the most widely used cancer biomarker [24,25]. Among five exons of PSA, we detected seven SNPs (Figure 1B). Two patients expressed a T→G transversion in the first codon of PSA exon 2 (PSA-c.48T>G, 2/33, 6.06%). One patient expressed an A→C transversion in the third codon of PSA exon 2 (PSA-c.54A>C, 1/33, 3.03%). One patient expressed a C→T transition in the 13th codon of PSA exon 2 (PSA-c.84C>T, 1/33, 3.03%). Seven patients expressed a C→T transition in the 11th codon of PSA exon 3 (PSA-c.237C>T, 7/33, 21.21%). These four SNPs were synonymous coding mutations and as such did not encode for a change in the amino acid sequence of PSA protein. However, we also detected three SNPs of a non-synonymous nature. Two patients expressed a G→A transition in the 34th codon of PSA exon 3 resulting in a D102N amino acid change (PSA-c.304G>A, 2/33, 6.1%). Four patients expressed a C→A transversion in the 64th codon of PSA exon 3 resulting in an L132I amino acid change (PSA-394C>A, 4/33, 12.1%). Two patients expressed a T→C transition in the 15th codon of PSA exon 4 resulting in an I179T amino acid change (PSA-c.536T>C, 2/33, 6.1%). Thus, albeit in a small patient population, amino acid divergent allelic variants of PSA, a smaller protein, were more common than those of our target antigen, PAP.

### Transcript variants of PAP are detectable by RTPCR from patient PBMC samples

**Figure 2:**
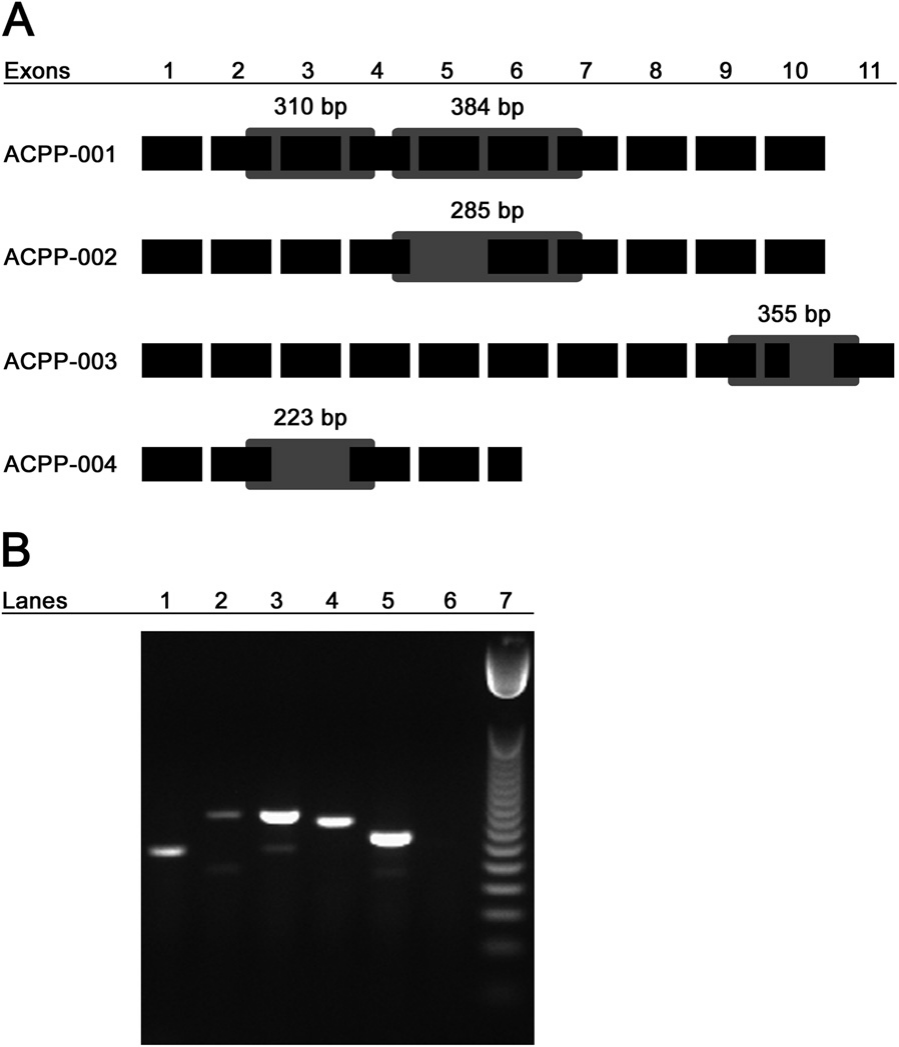
**Native and splice variant mRNA transcripts of PAP are detectable in patients prior to vaccination.** Panel **A**: Shown schematically are the four protein-coding transcripts of PAP including exons which are retained or excluded. Primer sets for ACPP-002 and ACPP-004 were designed to yield a native PAP product of indicated size as well as, if present, a variant PAP product of indicated size and as indicated by the gray boxes. Primer set for ACPP-003 was designed to yield a PAP-TM product of indicated size specific to the transmembrane domain-coding region of that transcript. Panel **B**: A representative agarose gel image of RTPCR products shows β-actin in lane 1, KLK3 (PSA) in lane 2, ACPP-002 native and variant bands in lane 3, ACPP-003 in lane 4, ACPP-004 native and variant bands in lane 5, no template control in lane 6, and 50 bp DNA ladder in lane 7.

Although we found that all patients receiving antigen-specific vaccination shared complete protein homology with the immunizing vector encoding PAP, messenger RNA transcripts exist for the native protein as well as eight other alternative splice variants. In total, there are four known protein-coding transcripts of PAP including ACPP-001 (native PAP), ACPP-002, ACPP-003, and ACPP-004 [26]. We hypothesized that expression of PAP alternative splice variants could present an additional set of antigenic epitopes not encoded by vaccine and, if expressed preferentially, may render immune responses to vaccine irrelevant and inform why immune response is not necessarily equivalent to clinical response following antigen-specific vaccination. We consequently sought to determine whether we could detect native and/or splice variant transcripts of PAP in our patients. Without an available resource of prostate or tumor biopsies from these patients, we collected mRNA from patient PBMC samples collected prior to vaccination and performed RTPCR. To detect the PAP variants ACPP-002 and ACPP-004, we designed primers that would yield a native PAP product as well as, if present, a variant PAP product of a smaller size (Figure 2A). To detect ACPP-003, we designed primers that would yield an amplicon specific to the transmembrane domain without amplifying native PAP. Figure 2A depicts the eleven pertinent exons of the four known protein-coding transcripts of PAP, shows the splice variant specific alterations for each member, and indicates the expected sizes of amplified regions using the primers designed (listed in Additional file 2: Table S2).

Following RTPCR amplification of mRNA from 27 patients with pre-vaccination PBMC still available, we found that detection of these four protein-coding transcripts was common in non-responding as well as immune-responding patients (Figure 2B and Table 3). Direct sequencing of isolated bands confirmed that RTPCR amplified expected transcripts of native PAP and splice variants of PAP (data not shown). We detected PSA transcripts in some prostate cancer PBMC samples (Table 3) but not in PBMC samples from volunteer blood donors (n=3, data not shown). Surprisingly, we did detect native PAP and/or one of the splice variants in three of three PBMC samples from patients without prostate cancer (data not shown). These results warrant further investigation into the transcriptional regulation and tissue-specificity of native PAP and splice variants; future studies are planned to expand upon these results. Nonetheless, the presence of detected alternative splice variants in patients with and without immune responses elicited to the native protein suggested that immune response to antigen-specific vaccination is neither hindered nor enhanced by their presence. As such their presence does not represent a means of circumventing immune responses to the native antigen.

**Table 3:** mRNA transcripts of native and splice variants of PAP are detectable in pre-vaccination patient PBMC

		**PAP mRNA transcripts**				**Control mRNA transcripts**	
	**Patients**	**ACPP-001**	**ACPP-002**	**ACPP-003**	**ACPP-004**	**β-actin**	**KLK3-201**
	1	**X**		**X**		**X**	**X**
	2	**X**	**X**	**X**	**X**	**X**	**X**
	3	**X**	**X**	**X**	**X**	**X**	
	4	**X**	**X**	**X**		**X**	**X**
	5					**X**	**X**
	6	**X**	**X**	**X**	**X**	**X**	
Non-Responders	7	**X**	**X**	**X**		**X**	
	8	**X**		**X**		**X**	**X**
	10			**X**		**X**	**X**
	11	**X**	**X**	**X**	**X**	**X**	**X**
	12	**X**		**X**	**X**	**X**	**X**
	13					**X**	
	14	**X**	**X**	**X**		**X**	**X**
	15	**X**		**X**	**X**	**X**	**X**
	16	**X**		**X**		**X**	**X**
	17	**X**	**X**	**X**	**X**	**X**	**X**
	18	**X**		**X**		**X**	**X**
	19	**X**	**X**	**X**		**X**	**X**
	24	**X**				**X**	**X**
Immune Responders	25	**X**				**X**	
	26					**X**	**X**
	27	**X**	**X**	**X**	**X**	**X**	**X**
	28	**X**		**X**		**X**	**X**
	29	**X**		**X**	**X**	**X**	**X**
	31	**X**		**X**		**X**	**X**
	32	**X**		**X**		**X**	**X**
	33	**X**	**X**	**X**	**X**	**X**	**X**

Shown are native and alternative splice variant PAP products detected among immune non-responding patients (top, 1–13) and immune responding patients (bottom, 14–33). Detection of an mRNA transcript is indicated by an X.

### Immune responses to regions specific to PAP splice variants are detectable in patients prior to vaccination with antigen-specific DNA vaccination

**Figure 3:**
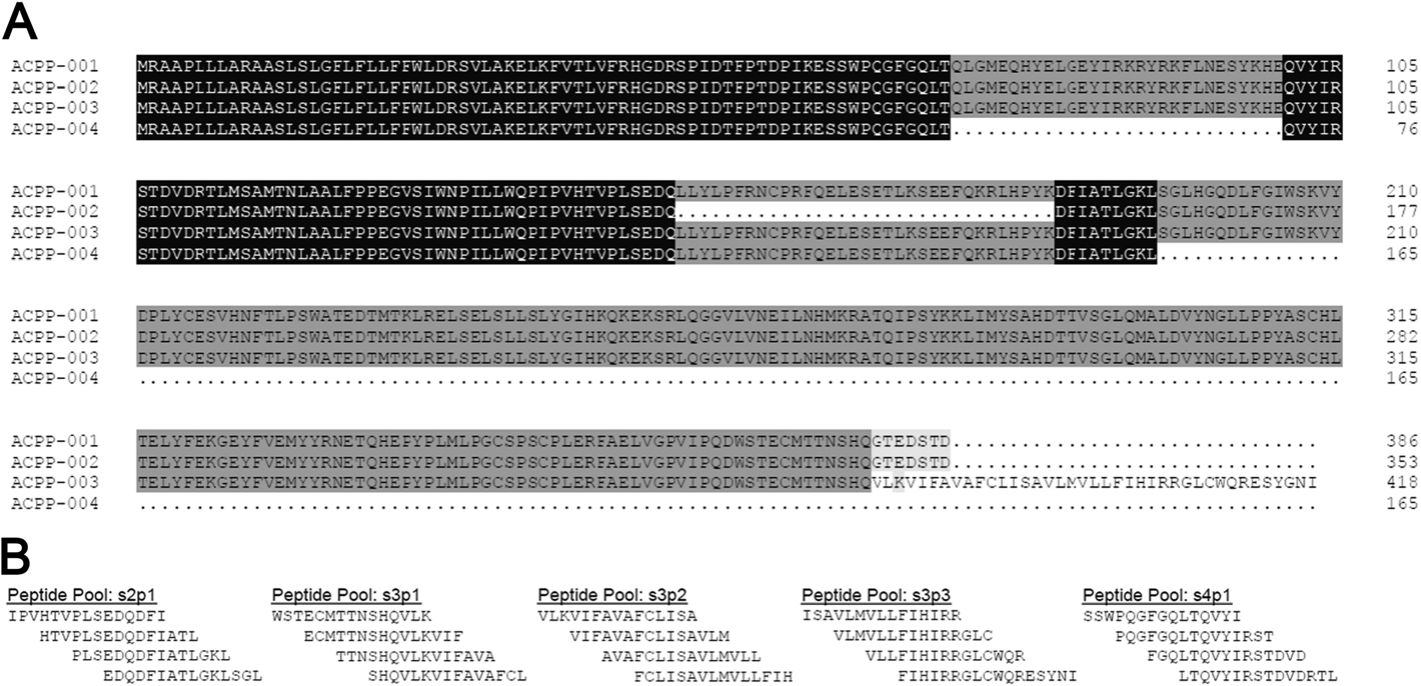
**Peptides specific to PAP splice variant proteins designed for the detection of pre-existing immune responses span splice junctions of variant proteins.** Panel **A**: Shown is an amino acid sequence alignment of the four protein coding alternative splice variants of PAP with the native PAP protein. Panel **B**: Shown are the amino acid sequences of five peptide pools spanning the alternative splice variants of PAP, including three pools for the transmembrane domain of ACPP-003.

We have observed PAP-specific immune responses in patients prior to vaccination that were augmented following antigen-specific vaccination [14]. In addition, we have previously reported that PAP-specific T-cell proliferative immune responses are detectable in ~11% of patients with prostate cancer irrespective of immunization [27]. In the current study, because patients expressed detectable transcripts of native and splice variant PAP, and in particular because expression of the splice variants was quite common among patients tested, we questioned whether patients who possessed detectable native PAP and/or PAP splice variant transcripts might exhibit immune responses to peptide epitopes specific to PAP splice variants. As such, to identify immune responses specific for the native or splice variants of PAP, we designed 15-mer peptide pools specific to the splice junctions of each alternative splice variant PAP protein (Figure 3A and B) and used a peptide pool comprised of 38 individual 15-mer overlapping peptides spanning the full length of the native PAP protein to identify immune responses to the native protein (ACPP-001) [28]. Among 27 patients tested for IFN-γ-secreting immune responses to alternative splice junction peptide pools from samples collected prior to clinical vaccination, we found evidence of immune response to the native PAP pool (PAP_1-386_) in 2 patients, similar to our previous report [27]. Responses to most splice variants were at a similar frequency, however responses to a portion of the transmembrane ACPP-003 (s3p2 pool) were detected at a high frequency, in 15 (56%) patients (Table 4). There was no difference in the frequency of this baseline response between immune responders and non-responders (*p=1.0*, Fisher’s exact test). Overall these results suggest that alternative splice variants of PAP are detectable in patients, some patients possess baseline immune responses to regions specific to those variants as well as to the native PAP, and immune responses to the transmembrane transcript variant of PAP were particularly common in these patients with prostate cancer. While these results suggest that the transmembrane variant of PAP might be particularly immunogenic, the presence of immune responses specific to the transmembrane transcript did not preclude the development of immune responses to the native PAP protein following immunization.

**Table 4:** Immune responses to peptides specific to PAP splice variant proteins are detectable in patients

		**Library**	**Splice junction peptide pools**					**Control antigens**		
**Patients**		**hPP**	**s2p1**	**s3p1**	**s3p2**	**s3p3**	**s4p1**	**rPP-100**	**SSX2-p167**	**PHA**
	1			**X**	**X**		**X**			**X**
	2		**X**	**X**	**X**		**X**			**X**
	3				**X**					**X**
	4								**X**	**X**
	5									**X**
	6									**X**
Non-responders	7									**X**
	8									**X**
	10				**X**					**X**
	11				**X**					**X**
	12				**X**					**X**
	13									**X**
	14				**X**					**X**
	15	**X**			**X**					**X**
	16					**X**				**X**
	17								**X**	**X**
	18	**X**	**X**	**X**			**X**	**X**	**X**	**X**
	19								**X**	**X**
	24									**X**
Immune responders	25				**X**					**X**
	26				**X**					**X**
	27				**X**					**X**
	28				**X**					**X**
	29			**X**	**X**					**X**
	31				**X**					**X**
	32				**X**					**X**
	33									**X**

PBMC from immune non-responding patients (top, 1–13) and immune responding patients (bottom, 14–33) obtained prior to immunization were assessed for IFN-γ-secreting immune responses by ELISPOT following stimulation with peptides pools specific to the alternative splice variants of PAP or a peptide pool from the native antigen. The presence of a statistically significant IFN-γ-secreting immune response to a specific peptide pool as described above is indicated by an X.

## Discussion

At the outset of this investigation we questioned whether differences in immune response following antigen-specific vaccination might be due to a phenomenon similar to xenogeneic cross-reactivity. Specifically, we hypothesized that patients expressing highly homologous allelic variants and/or alternative splice variants of PAP might present different epitopes than those encoded by the vaccine, permitting the vaccine to invoke a cross-reactive response to homologous epitopes. This seems to not be the case for these patients and this antigen. However, we believe these findings are novel and relevant to the tumor immunology field in that: 1) this is the first examination of the allelic variation of a somatic gene targeted by genetic vaccination among patients receiving said vaccine; 2) this is the first report showing detection of mRNA transcripts of native PAP and alternative splice variants of PAP from peripheral blood samples; 3) this is the first evaluation of associations between immune response to vaccination and the detection of allelic or transcriptional antigen variants; and 4) this report effectively demonstrates in humans that antigen-specific immunization can elicit a response to the host genome-encoded antigen. In addition, our report demonstrates that IFN-γ-secreting immune responses specific for alternative splice variants of PAP are common, suggesting that, in particular, the transmembrane variant of PAP might be a relevant immunological target antigen for future investigation.

We detected synonymous coding mutations of PAP in the somatic DNA of 33 patients that went on to receive a genetic vaccine encoding an antigen with an amino acid sequence identical to their own (pTVG-HP). Our initial interest was that genetic variation in the target antigen could predict or inform immunological response to subsequent vaccination. We have previously published results indicating that some patients developed immune responses following vaccination with pTVG-HP and others did not, and the present sequencing results suggest that detectable immune responses were likely reactive to the PAP self-antigen in contrast to other vaccines which take advantage of xenoantigen cross-reactivity to elicit responses [13,14]. While this is the probable case for these patients immunized with PAP-targeted vaccines, immunogenicity and frequency of allelic variation of other vaccine antigens (such as gp100, PSMA, PSA and tyrosinase) may differ greatly from those known presently. More to the point, these results suggest that vaccination with pTVG-HP, or sipuleucel-T which similarly targets PAP, are truly targeting the antigen of interest, rather than a highly homologous or “foreign” protein. However, it should be noted that we examined the expression of allelic variation in somatic DNA coding for the target antigen. PAP protein expression can increase with progression of prostate cancer, being expressed on AR-negative neuroendocrine cells, and it is thus possible that due to an evolving karyotype, genetic mutation of proteins expressed by tumor cells (not detectable in our analysis) could allow for immune evasion following vaccination [29]. In the present study, patient tumor samples were not available to evaluate the association between PAP mutations in tumor cells and immune response to PAP-specific vaccination. Moreover, the trials from which these samples were obtained accrued patients with minimal residual disease, without radiographic evidence of metastases, hence such samples were not available. Certainly, future studies examining differences in immune response to vaccination with respect to possible variations in tumor-encoded antigen could be informative.

As shown in Table 1, PAP is one of the more highly conserved vaccine target antigens currently under investigation. PSA is less conserved, with a greater number of protein-altering mutations previously detected, despite it being a protein of fewer amino acids. While we detected non-synonymous coding mutations of PSA we cannot know the impact this may (or may not) have on the efficacy of a genetic vaccine encoding PSA in these patients. As these allelic variants encode for an amino acid sequence different from the native PSA protein, broader investigation of variants of this antigen is warranted [30,31]. PROSTVAC™ is a genetic vaccine encoding PSA currently being investigated in Phase III clinical trials, and with such a large patient population associations of immunological and/or clinical response with expression of non-synonymous coding mutations of PSA might be possible. Serum PSA protein replaced PAP as the standard clinical biomarker for the detection and diagnosis of prostate cancer, as well as for staging and monitoring prostate cancer progression [32,33]. Both of these prostate-specific serum proteins, PAP and PSA, are measured in the serum of patients with prostate cancer, using immunometric assays specific to each protein [34]. Immunometric assays allow sufficient sensitivity for the detection of small quantities of secreted protein as biomarkers. However, this assay utilizes antibodies that recognize specific epitopes of the protein to be measured. In patients expressing non-synonymous coding mutations or alternative splice variants within recognized epitopes which alter antibody binding, detection and measurement of serum protein could be compromised.

In the present study, we did not observe differences between immune non-responding and immune responding vaccine patients in their expression of allelic variants of PAP or in their expression of alternative splice variants of PAP. A major goal for investigators in the field of cancer immunotherapy is the identification of patient characteristics capable of predicting immune response and/or informing vaccine target selection [35]. While we did not find evidence of response discrimination, larger patient population and inclusion of other somatic and tumor antigens in the sequencing step might aid in this goal. We found a significant association between immune response to pTVG-HP and the synonymous mutation: PAP-c.122T>G. Others have published interesting, sometimes deleterious, effects of synonymous coding mutations including alteration of spliceosome recognition sites, activation of cryptic splice donor sites, destabilization of mRNA, alteration of codon preference during splicing, modulation of mRNA degradation, and/or ribosomal interaction during translation [36-39]. This particular mutation occurs within the first codon of the second exon. Hence, it is conceivable that this variant affects correct splicing and thus might affect antigen expression overall rather than affect a specific epitope. We were not able to evaluate PAP protein expression in individual subjects, however the possibility that synonymous mutations could lead to difference in protein expression that affects immunological response provides an avenue for future studies. In addition, expanding the patient population and perhaps including upstream promoter regions, intronic and downstream regulatory regions, and androgen- or immune-related genes as well as evaluating the possible structural and interacting elements influencing this antigen are also of great future interest.

Other criteria have been reported or discussed as possibly predicting for immune response to vaccination, including the so-called “immune score,” pre-existing immune responses, and other intrinsic patient characteristics [40-42]. It is hypothesized that DNA vaccination is facilitated by TLR9 activation by vaccine-encoded CpG motifs. Of interest, Silver and colleagues reported that TLR9 expression and function demonstrate responsiveness to circadian rhythm in mice [43]. As such, it is conceivable that the timing of DNA vaccination in human patients might be scheduled to increase immunogenicity. In addition, recent work from our lab using samples from some of these same patients, showed that PAP-specific T-regulatory cells expressing CTLA-4 and secreting IL-35 exist in some individuals prior to vaccination and persist during immunization [44]. This suggests that the detection of antigen-specific regulatory T-cell responses might be an additional means of identifying patients likely to respond, or not, to vaccination.

The present study examines baseline genomic, transcriptional, and immunological differences in a protein target antigen (PAP) against which study patients eventually were vaccinated with a genetic vaccine. The field of cancer immunotherapy continues to make progress towards elucidating the reasons or mechanisms for why one patient responds and another does not. This study may represent a starting point for future workflow methodologies attempting to “personalize” antigen-specific immunization to identify which patients are most likely (or not) to respond to immunization.

## Conclusions

In this report we hypothesized that patients expressing highly homologous allelic variants and/or alternative splice variants of a tumor antigen (PAP) might present different epitopes than those encoded by a genetic vaccine, thus permitting the vaccine to invoke a cross-reactive response to homologous, but potentially not “self,” epitopes. Despite a relatively small population, we did not identify non-synonymous amino acid coding variants within the genomic sequences encoding this antigen, however expression of alternative PAP splice variants was common. Neither was associated with immune outcome, effectively demonstrating that genetic vaccination can elicit a response to the host genome-encoded antigen and that immune responses detected are not directed to “foreign” epitopes presented during vaccination. We believe these findings are of relevance to other anti-tumor vaccines, including sipuleucel-T, the only FDA-approved anti-tumor vaccine that similarly targets the PAP tumor antigen.

## Methods

### Patient population, study agent, regulatory information, and clinical trial information

We have previously reported the results of a Phase I trial using a plasmid DNA vaccine encoding human PAP, pTVG-HP, in patients with biochemically (serum PSA) recurrent prostate cancer after definitive surgery and/or radiation therapy not receiving androgen deprivation therapy [13,14]. A similar Phase Ib/II trial using pTVG-HP is ongoing at the time of this writing, in patients with biochemically (serum PSA) recurrent prostate cancer after definitive surgery and/or radiation therapy, without radiographic evidence of metastases, and receiving androgen deprivation therapy (NCT00849121). Cryopreserved peripheral blood mononuclear cell (PBMC) samples from these two trials were prepared using the same procedures, and used for the analyses described. In short, PBMC from both clinical trials were purified from blood in heparinized collection tubes generally within 6 hours of phlebotomy appointment and were cryopreserved in liquid nitrogen. Samples were grouped as immune responders and immune non-responders based on previous detection of PAP-specific interferon-gamma secreting immune response up to one year following vaccination with pTVG-HP by ELISPOT, as previously described [14]. Specifically, patients with significant (p < 0.05 by two-tailed *t* test comparison with media-only control) PAP protein-specific IFNγ response detectable at two or more time points over one year following vaccination were designated as immune responders. Patients not meeting these criteria were designated immune non-responders. Multiple vials from each patient in either response pool were effaced to remove identification and then recoded numerically to maintain anonymity. Study procedures were reviewed and approved by the Institutional Review Board of the University of Wisconsin-Madison.

Written informed consent was obtained from all patients for use of blood samples for research remaining from the clinical trials, and the specific research of this report was approved by the IRB of the University of Wisconsin-Madison.

### Genomic DNA extraction, primer design, PCR amplification, and direct Sanger sequencing

Somatic, genomic DNA was extracted from cryopreserved PBMC samples using Wizard Genomic DNA Extraction Kit (Promega, Madison, WI, USA) per the manufacturer’s instructions. DNA samples were quantified by Nanodrop spectrophotometer (Thermo Fisher Scientific, Wilmington, DE, USA). PCR amplification and sequencing genomic DNA oligonucleotide primers were designed for ten exons of PAP (ACPP-001, Ensembl Gene ID: ENSG00000014257) and five exons of PSA (KLK3-201, Ensembl Gene ID: ENSG000000142515) from genomic sequence data available (Additional file 1: Table S1). Samples were sequenced at the University of Wisconsin Biotechnology Center DNA Sequencing Core Facility using described primers by the direct Sanger method [45]. Sequence data were retrieved for all 15 exons from 33 patients including 5’ and 3’ sequence runs. Tracings were viewed with FinchTV 1.3.1 (Geospiza, Seattle, WA, USA) and chromatograms were exported, as is, to FASTA sequence file format. FASTA DNA sequences were analyzed with DNAMAN 5.2.8 (Lynnon Corp, Pointe Claire, Quebec, Canada). Multiple sequence alignments were created from all patients for each sequence in both 5’ and 3’ directions, referenced to expected sequences taken from the Ensembl database. Consistency and fidelity were cross-referenced against original tracing data and compared between 5’ and 3’ sequencing runs for each patient exon.

### RNA purification, cDNA synthesis, and reverse-transcriptase PCR

Messenger RNA was purified from PBMC using Qiagen RNeasy Mini Kit (Valencia, CA, USA) according to the manufacturer’s instructions. RNA samples were quantified by Nanodrop spectrophotometer. RTPCR using the Qiagen One-Step RT-PCR Kit was performed as previously described using collected mRNA from patient samples and primers indicated in Additional file 2: Table S2 [46]. Briefly, the following reaction conditions were used: 50°C for 30 minutes, 95°C for 15 minutes, 35 cycles of 95°C for 1 minute, specific annealing temperature of 60°C for 1 minute, and 72°C for 1 minute, and a final extension of 72°C for 10 minutes. Reaction products were then separated and evaluated by agarose gel electropheresis. Amplified product bands were isolated from agarose gel using Qiaquik Gel Extraction Kit (Qiagen) and collected DNA samples were sequenced for confirmation.

### Peptides

Four protein-coding transcripts of PAP were aligned by amino acid sequence using DNAMAN 5.2.8: ACPP-001 (native PAP, Ensembl Transcript ID: ENST00000336375), ACPP-002 (ENST00000475741), ACPP-003 (ENST00000351273), and ACPP-004 (ENST00000495911). 15-mer peptides spanning splice junctions of the alternative PAP variants were synthesized (Lifetein, South Plainfield, NJ, USA), and were >85% in purity confirmed by high-pressure liquid chromatography (HPLC) and mass spectroscopy (MS). A peptide pool (PAP_1-386_) comprised of 38 individual 15-mer overlapping peptides spanning the full length of the native PAP protein (ACPP-001) was used to identify immune responses to conserved regions of PAP [28]. Control peptides included a 15-mer peptide (QVYIRSTDVDRTLMS) derived from the rat PAP homologue and a 9-mer peptide (RLRERKQLV) derived from the human SSX-2 protein [47].

### Interferon-gamma ELISPOT

Immune response to antigen stimulation was determined by 48-hour interferon-gamma (IFN-γ) ELISPOT assay (Thermo Fisher Scientific), using similar methodology and test antigens as previously described [14]. PBMC at 100,000 cells per well were incubated with 2 μg/μL alternative splice variant peptide pools, 2 μg/μL control peptides, 1 μg/μL PAP_1-386_, and 5 μg/μL phytohemagglutinin (PHA) in eight-well replicates. The number of spots per well was determined with an automated ELISPOT reader (AID Diagnostika GmbH, Straussberg, Germany) and normalized to 10^6^ starting PBMC. For each subject, the mean number of spots detected under media-only conditions was subtracted from the antigen-specific conditions to enumerate antigen-specific IFN-γ spot-forming units ± standard deviation. For all patients the median number of IFN-γ spot-forming units with medium alone was 104 (range 9–2408) per million PBMC. Comparison of experimental wells with control media-only wells was performed using a two-tailed *t* test, with *p*-value less than 0.05 used to define a significant T-cell response.

## Abbreviations

Aa: Amino acid; CEA: Carcino-embryonic antigen; CTL: Cytotoxic T-lymphocyte; HPLC: High-pressure liquid chromatography; MS: Mass spectrometry; NSCLC: Non-small cell lung cancer; PAP: Prostatic acid phosphatase; PBMC: Peripheral blood mononuclear cells; PHA: Phytohemagglutinin; PSA: Prostate-specific antigen; PSMA: Prostate-specific membrane antigen; SNP: Single nucleotide polymorphism; TAA: Tumor-associated antigen.

## Competing interests

The authors declare no conflict of interest.

## Authors' contributions

JTB carried out the sequence analysis and experimental plan, and was primarily responsible for the interpretation and analysis of results and manuscript preparation. DGM conceived of the study, and participated in the design and coordination, and helped to draft the manuscript. Both authors read and approved the final manuscript.

## Supplementary Material

Additional file 1: Table S1 Genomic DNA primers for amplification and sequencing of PAP and PSA exons. Click here for file

Additional file 2: Table S2 DNA primers for amplification and sequencing of native and/or alternative splice variants of PAP. Click here for file
