# Supplementary material for: Presence of antigen-specific somatic allelic mutations and splice variants do not predict for immunological response to genetic vaccination
Source: J Immunother Cancer. 2013 May 29;1:2. doi: 10.1186/2051-1426-1-2 (PMC3986973; doi:10.1186/2051-1426-1-2)
Supplement: Additional file 1: Table S1 — Genomic DNA primers for amplification and sequencing of PAP and PSA exons. [file 2051-1426-1-2-S1.pdf]

Additional file 1: Table S1: Genomic DNA primers for amplification and sequencing of PAP and PSA exons.

**Prostatic Acid Phosphatase (PAP)**		
Exon	5’	3’
1	CAT ACC AAA CCT GCC CTA CG	TGG TCT CTG GGA AAC AAT CC
2	GGT TGA AAG CCA GTC CTC AG	CCA CAC CCA GCT TCA TCA ACA ATC
3	CAA TCA CCC AAC TAT CAA CTA AGC AC	CAA GAC CTC TGA CTT GTA TAA GTT CC
4	TGT GGG TGT CCT TTC CTT TC	TGC ACA GCT CAA GGA GTG TC
5	TTT CCA CTC AGC TCA AAA CAC	TGC TGT AAC CAG CCA CTG AC
6	GTG AAA CAT CCT ATA ATG CAC AGG AG	GAG AAA AGA GAA GTC AGC ATT TTC AAG
7	GGC AGG ACA AGA AAG TCT GG	AGA ATG GAA GCT GGT GCA AG
8	AGG GAG AGT CCG CAA CTA TG	CTT GCC TGC ATG GTA TTT CC
9	CCA CGC CCA GCC AAT TTC TTC ATC	CAG GCA CAC AAC CCT TAA CC
10	CAT TTC CAC CCT GCA AAT CTC TCC C	AAA GCA TCA TCT GCC CTG AG
**Prostate-Specific Antigen (PSA)**		
Exon	5’	3’
1	CCA GGG TGA TCT AGT AAT TGC AGA AC	GAG TGA GGA CTG TTG CAA TAT GGA G
2	GTC TCC TAC CCT GAT CCC TGG GTT C	GTG ACC TGA ACC AGA ACT TTC CCT C
3	CCT TAT CAT CCT CGC TCC TCA TTC C	CAG GCA TCT GGG CTC CCG GCT GCA C
4	GTC CAG CCC ACA ACA GTG TTT TTG	GAA GAG GGA AGG AGG GAA TGA GAT G
5	CTC ACT CTC TCC CTG CTT TTA CCC	GGA CAC AGA GAG GAC AAA ATT ACA CC
