# Supplementary material for: Presence of antigen-specific somatic allelic mutations and splice variants do not predict for immunological response to genetic vaccination
Source: J Immunother Cancer. 2013 May 29;1:2. doi: 10.1186/2051-1426-1-2 (PMC3986973; doi:10.1186/2051-1426-1-2)
Supplement: Additional file 2: Table S2 — DNA primers for amplification and sequencing of native and/or alternative splice variants of PAP. [file 2051-1426-1-2-S2.pdf]

Additional file 2: Table S2: DNA primers for amplification and sequencing of native and/or alternative splice variants of PAP.

**PAP mRNA Transcript Variant Primers**		
Variant	5'	3'
ACPP-002	CGA AGC ACA GAC GTT GAC CGG AC	CAT GGT GTC CTC AGT GGC CCA G
ACPP-003	CTG TGA GTG GCC TAC AGA TGG C	CAG CAG AGT CCA CGG CGA ATG
ACPP-004	CGG CAT GGA GAC CGA AGT CCC	CTG TGT GCA CCG GGA TGG GC
**Control mRNA Transcript Primers**		
Pair	5'	3'
KLK3-201	CCA GCC ACG ACC TCA TGC TGC T	CTT GGT CAC CTT CTG AGG GTG AAC
β-actin	TCA TGA AGT GTG ACG TTG ACA TCC GT	CTT AGA AGC ATT TGC GGT GCA CGA TG
